# Pulmonary tuberculosis with false-positive ^18^F-fluorodeoxyglucose positron emission tomography mimicking recurrent lung cancer: A case report

**DOI:** 10.3892/etm.2014.2054

**Published:** 2014-11-07

**Authors:** CHENG CHEN, YE-HAN ZHU, HONG-YING QIAN, JIAN-AN HUANG

**Affiliations:** Respiratory Department, The First Affiliated Hospital of Soochow University, Suzhou, Jiangsu 215006, P.R. China

**Keywords:** tuberculosis, recurrence, lung cancer, positron emission tomography-computed tomography

## Abstract

Recurrent lung cancer is a common clinical condition. ^18^F-fluorodeoxyglucose positron emission tomography (FDG-PET) is currently the predominant non-invasive imaging technique used for the detection of tumor recurrence. In the present study, the case of a 67-year-old male suspected to have postoperative recurrence of primary lung cancer was examined. Chest computed tomography (CT) scans identified a subpleural nodule grown within a short time period, along with the occurrence of multiple patchy shadows on the right lung. PET-CT scans revealed an increased FDG uptake in the surgical site, which exhibited features of a malignant disease. However, a video-assisted thoracoscopic biopsy provided the diagnosis of tuberculosis and guided further appropriate treatment. In conclusion, further evaluation is required in all patients with suspected metastatic and recurrent carcinoma.

## Introduction

Treatment of lung cancer usually involves surgery in the early stages and radiotherapy or chemoradiotherapy in more advanced stages of the disease (1). Cancer recurrence may occur in multiple sites following primary treatment. Although recurrent and metastatic disease is usually not curable, surgical treatment may be beneficial in cases where locoregional recurrence is detected early. Performing ^18^F-fluorodeoxyglucose positron emission tomography-computed tomography (^18^F-FDG PET-CT) is important in the diagnosis of clinically suspicious recurrent lung cancer (2). However, the diagnostic efficiency of PET-CT remains controversial (3,4). A previous study demonstrated that delayed PET-CT scans enhanced the variation of FDG uptake between a number of lesions (5). In addition, delayed maximum standardized uptake values (SUVmax) of >5.5 were shown to improve the differentiation of hypermetabolic lesions compared with earlier scans. However, careful interpretation and management are still required for correct diagnosis. In the present study, a case of pulmonary tuberculosis, suspected as recurrent lung cancer following PET-CT scans, was examined.

## Case report

**Figure 1:**
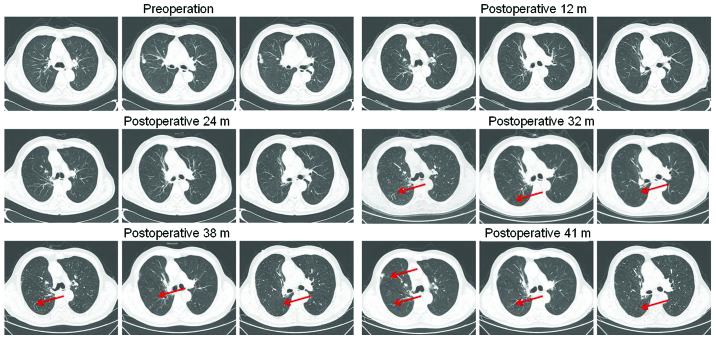
Computed tomography (CT) scans of the patient. No evident changes were observed in the chest radiographs until 32 months following surgery, when multiple patchy shadows were observed on the right lung. After a further nine months, chest CT scans revealed a subpleural nodule (0.5×0.5 cm) and a slight enlargement of the patchy shadow. m, months.

**Figure 2:**
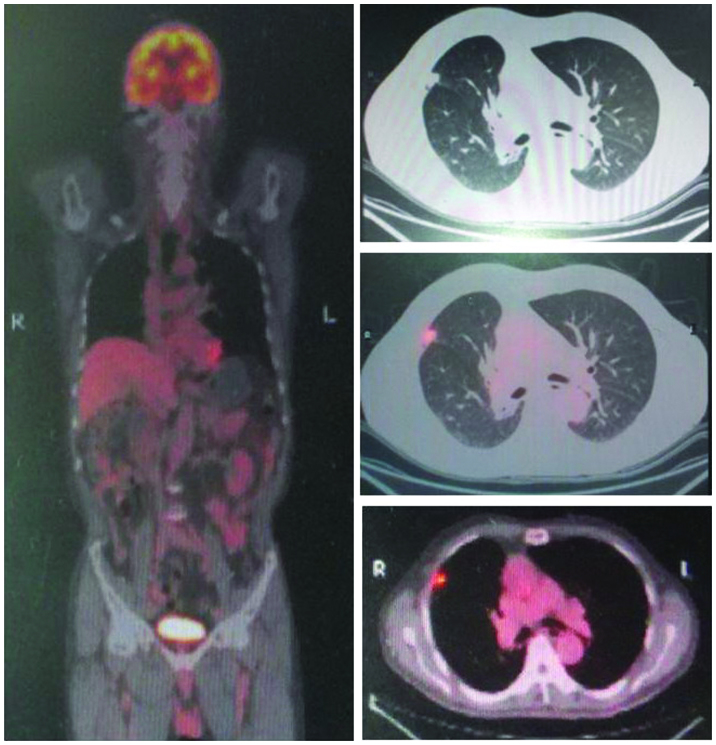
Positron emission tomography-computed tomography scans showing an abnormality with increased ^18^F-fluorodeoxyglucose uptake at the surgical site.

**Figure 3:**
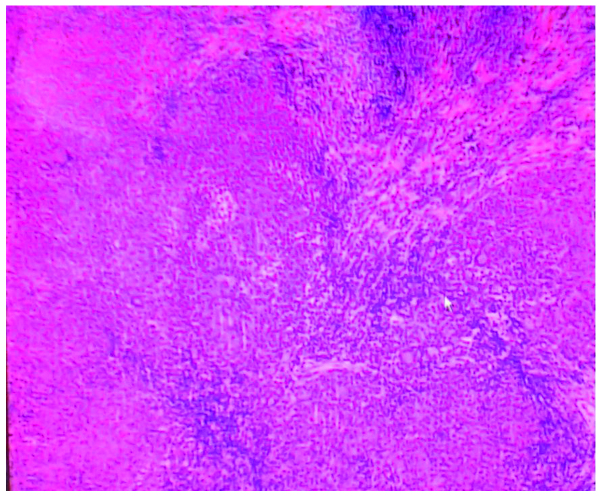
Hematoxylin and eosin staining of the tissue, showing the presence of a granuloma and caseous necrosis (magnification, ×200).

A 67-year-old male was admitted to the Surgical Outpatients Department of The First Affiliated Hospital of Soochow University (Suzhou, China), with a mass lesion (2×2 cm) on the right lung (Fig. 1). The lesion was detected during a routine medical examination. A video-assisted thoracoscopic lobectomy was performed to remove the mass, and histopathological examination revealed a lung adenocarcinoma with visceral pleural invasion. The patient underwent four courses of adjuvant chemotherapy (75 mg/m^2^ cisplatin and 100 mg/m^2^ gemcitabine) and was clinically diagnosed as disease free. However, the appearance of multiple patchy shadows on the right lung was observed in CT scans at 32 months following the surgery (6). The patient did not present any evident changes in the chest radiographs and serum tumor marker tests during the subsequent nine-month follow-up period. After the nine-month period, chest CT scans revealed a subpleural nodule (0.5×0.5 cm) and a slight enlargement of the patchy shadow on the right lung (Fig. 2). PET-CT scans revealed that the FDG uptake of the nodule had a SUVmax of 6.1. A that indicated malignant disease. A video-assisted thoracoscopic biopsy indicated that the patient suffered from tuberculosis (Fig. 3). Written informed consent was obtained from the patient.

## Discussion

Adenocarcinoma is the predominant histological subtype of lung carcinoma (7); however, smear pulmonary tuberculosis appearing on the surgical site as the recurrence of lung cancer is extremely rare, particularly during the postoperative follow-up period.

Postoperative recurrence of lung cancer is commonly diagnosed by CT or PET-CT scans and clinical features, instead of a rebiopsy. PET-CT is the most sensitive non-invasive imaging method for the detection of tumor metastases and recurrence, since these scans enable more accurate assessment of the tumor morphology, composition, location and extent (8). However, the diagnostic efficiency of PET-CT remains controversial, since a number of benign lesions may exhibit increased metabolic activity, leading to a false positive result. Failure to differentiate between recurrent tumors and benign lesions may result in inappropriate treatment. Therefore, previous studies have attempted to evaluate the accuracy of PET-CT as a diagnostic method, and the characteristics of false negatives and false positives, to improve specificity and sensitivity (5,9). Razak *et al* demonstrated that early whole body PET-CT may efficiently detect extrapulmonary tuberculosis lesions, while dual time point imaging may not be able to determine the lesion type (9).

The present study reports the case of a 67-year-old male with pulmonary tuberculosis, whose PET-CT images mimicked recurrent lung cancer. Due to the slow progress of the multiple patchy shadows on the right lung, along with the increased FDG uptake in a short-term growing subpleural nodule, the patient was suspected to suffer from recurrent lung cancer. However, the slow progress of the multiple patchy shadows and the normality of the serum tumor markers prompted a video-assisted thoracoscopic rebiopsy to be performed. Pathological examination confirmed the inference that the subpleural nodule was due to tuberculosis instead of recurrent lung cancer. Therefore, further evaluation is required in all patients with a suspected metastatic and recurrent carcinoma, and rebiopsy is a valuable method for certain patients, since other conditions may exist, including a benign disease.
